# Genomic Analysis by Deep Sequencing of the Probiotic *Lactobacillus brevis* KB290 Harboring Nine Plasmids Reveals Genomic Stability

**DOI:** 10.1371/journal.pone.0060521

**Published:** 2013-03-27

**Authors:** Masanori Fukao, Kenshiro Oshima, Hidetoshi Morita, Hidehiro Toh, Wataru Suda, Seok-Won Kim, Shigenori Suzuki, Takafumi Yakabe, Masahira Hattori, Nobuhiro Yajima

**Affiliations:** 1 Research Institute, KAGOME Co., Ltd., Nasushiobara, Tochigi, Japan; 2 Graduate School of Frontier Sciences, The University of Tokyo, Kashiwa, Chiba, Japan; 3 School of Veterinary Medicine, Azabu University, Chuo-ku, Sagamihara, Kanagawa, Japan; 4 Medical Institute of Bioregulation, Kyushu University, Higashi-ku, Fukuoka, Japan; Baylor College of Medicine, United States of America

## Abstract

We determined the complete genome sequence of *Lactobacillus brevis* KB290, a probiotic lactic acid bacterium isolated from a traditional Japanese fermented vegetable. The genome contained a 2,395,134-bp chromosome that housed 2,391 protein-coding genes and nine plasmids that together accounted for 191 protein-coding genes. KB290 contained no virulence factor genes, and several genes related to presumptive cell wall-associated polysaccharide biosynthesis and the stress response were present in *L. brevis* KB290 but not in the closely related *L. brevis* ATCC 367. Plasmid-curing experiments revealed that the presence of plasmid pKB290-1 was essential for the strain's gastrointestinal tract tolerance and tendency to aggregate. Using next-generation deep sequencing of current and 18-year-old stock strains to detect low frequency variants, we evaluated genome stability. Deep sequencing of four periodic KB290 culture stocks with more than 1,000-fold coverage revealed 3 mutation sites and 37 minority variation sites, indicating long-term stability and providing a useful method for assessing the stability of industrial bacteria at the nucleotide level.

## Introduction

Probiotics are live microorganisms that, when administered in adequate amounts, confer a health benefit on the host [1]. Several microorganisms have probiotic properties, and those most commonly used are lactic acid bacteria, including lactobacilli. Over recent decades, as awareness of the beneficial effects of probiotics in promoting gut and general health has grown, the development and consumption of probiotic foods has increased worldwide [2]. Probiotics exhibit strain-specific differences in acid and bile resistance, ability to colonize the gastrointestinal (GI) tract, clinical efficacy, and the health benefits they confer. The genomes of many probiotic bacterial strains have been sequenced and analyzed in efforts to reveal the genes or metabolic pathways involved in their health-promoting traits [3]–[5].

*Lactobacillus brevis*, an obligate heterofermentative Gram-positive organism, is frequently found on plant materials as well as in other niches, including beverages and the human intestinal tract [6], [7]. Several *L. brevis* strains have been tested for probiotic qualities, some in clinical trials [8]–[10]. Genomic analysis of *L. brevis* ATCC 367 revealed several genes that likely contribute to its probiotic activity [3], [11], [12].

*L. brevis* KB290 was, another probiotic strain, originally isolated from suguki, a traditional Japanese fermented vegetable [13]. KB290 has been found to tolerate gastrointestinal juices, stimulate immune function [13]–[15], and improve gut health [13]–[15]. Moreover, KB290 has a strong tendency to aggregate in broth medium, which may be a desirable property that allows for co-aggregation with pathogenic bacteria, and it colonizes and immunomodulates colonic mucosa [16]–[21]. Because of these desirable traits, KB290 has been used in fermented food products in Japan since 1993, but little is known about its genetic structure.

Here we completely sequenced the KB290 genome and compared it with the genomes of other *Lactobacillus* strains to determine the basis of its unique probiotic traits. Using next-generation sequencing, we also performed deep sequencing of KB290 and old culture stocks to detect low frequency variants, thereby assessing genomic stability.

## Materials and Methods

### Bacterial strains and growth conditions

*L. brevis* KB290 was deposited by KAGOME Co., Ltd., as strain *L. brevis* JCM 17312 in the Japan Collection of Microorganisms. We derived plasmid-cured strains (KB2901, KB2902, and KB2903) from a KB290 stock culture that had been frozen since 2009 (KB290_2009) as previously reported [22], and obtained a spontaneous plasmid-cured strain (KB392) by cultivating KB290_2005 (frozen since 2005) in nutrient-limited medium. KB290 and three old stock strains—KB290_1994 (frozen since 1994), KB290_2005 (frozen since 2005), and KB290_2006 (frozen since 2006)—were subjected to deep sequencing as described below. Table 1 lists the bacterial strains used in this study. We confirmed that strains were plasmid-cured with the polymerase chain reaction (PCR) using the plasmid-specific primers listed in Table S1, PuReTaq Ready-To-Go PCR Beads (GE Healthcare), and a Bio-Rad MyCycler thermal cycler (Bio-Rad). All strains were grown in de Man Rogosa Sharpe (MRS) medium (Oxoid) at 30°C.

**Table 1:** *L. brevis* strains used in this study.

Strain	Description	Reference
KB290	KB290 stock-culture prepared in 2009	This study
KB290_1994	KB290 stock-culture prepared in 1994	[13]
KB290_2005	KB290 stock-culture prepared in 2005	This study
KB290_2006	KB290 stock-culture prepared in 2006	This study
KB392	Spontaneous plasmid-cured strain of KB290 (pKB290-1^−^, pKB290-2^−^, pKB290-3^−^)	This study
KB2901	Plasmid-cured strain of KB290 (pKB290-2^−^, pKB290-3^−^)	This study
KB2902	Plasmid-cured strain of KB290 (pKB290-3^−^, pKB290-4^−^)	This study
KB2903	Plasmid-cured strain of KB290 (pKB290-3^−^)	This study
ATCC 367	Previously sequenced strain	[3]

### DNA preparation

We prepared genomic DNA from late-logarithmic phase KB290 cells using standard genomic DNA affinity columns and isolated plasmid DNA as previously described [23], except that we preincubated the cells with 10 mg/mL lysozyme (Sigma) and 50 U/mL mutanolysin (Sigma) for 1 h at 37°C to weaken the cell wall prior to cell disruption.

### Genome sequencing

The KB290 genome was sequenced using the whole-genome shotgun strategy with a 3730xl sequencer (Applied Biosystems). About 20 μg of genomic DNA was sheared using a HydroShear (Gene Machines), and the DNA fragments were fractionated by agarose gel electrophoresis. Fractions were subcloned into the plasmid pTS1 vector (Nippon Gene) for construction of shotgun libraries with average insert sizes of 2 kb and 5 kb. Template DNA was prepared by PCR amplification of inserts of clones with Ex-Taq (Takara Bio) from an aliquot of the bacterial culture, and the DNA was subjected to Sanger sequencing, generating 34,560 reads (8.7-fold coverage) from both ends of the clones. Data were managed using Phred, Phrap and Consed [24], and gaps were closed by direct sequencing of clones that spanned the gaps or of PCR products amplified with oligonucleotide primers designed to anneal to each end of neighboring contigs. A finished sequence with an error rate of <1 per 10,000 bases (Phred score ≥40) was obtained.

### Deep sequencing of periodic KB290 strains

We performed deep sequencing of four periodic KB290 strains—KB290_1994, KB290_2005, KB290_2006, and KB290 (KB290_2009)—using 5500xl SOLiD (Life Technologies) using 120- to 500-ng genomic DNA sheared into 100- to 250-bp fragments using a Covaris S2 System. Both ends of the fragments were repaired, phosphorylated, and ligated with two types of adaptors (P1 and P2). The adaptor-ligated DNA fragments were selected by size for library construction, and the library was directly transferred to the nick translation reaction. After amplifying the libraries with Platinum polymerase (Invitrogen), we quantified the products by quantitative PCR and titrated the DNA concentration to achieve 10%–20% single template beads in the emulsion PCR using each 10-fmol library DNA. After successive bead enrichment and deposition of 600 million beads, sequencing was carried out using 2/6 slide and the mapped reads were generated as follows: 57,946,515 for KB290, 39,262,750 for KB290_1994, 53,561,444 for KB290_2005, and 54,076,405 for KB290_2006. These reads gave approximately 1,700-fold (KB290_2009), 1,100-fold (KB290_1994), 1,500-fold (KB290_2005), and 1,600-fold coverage (KB290_2006) for each isolate. The data were analyzed with the Bioscope alignment pipeline (Life Technologies) for mapping, and single nucleotide variants were called with the diBayes algorithm integrated into the Lifescope package (Life Technologies). We applied a bimodal strategy to detect variants having coverage ≥30-fold and a read frequency ≥20%.

### Sequence analysis

Protein-coding genes were identified with Glimmer3 [25]. Similarity searches of all the predicted proteins were performed against the non-redundant database/NCBI and Swiss-Prot/EMBL and the toxin and virulence factor database MvirDB [26] using BLASTP (E-value ≤10^−10^; identity ≥30%; coverage ≥30%). The tRNA genes were predicted using tRNAscan-SE [27] and were searched for bacterial RNA databases using BLASTN. Functional classification of protein-coding genes was performed using NCBI clusters of orthologous groups (COGs) [28] using BLASTP (E-value ≤10^−10^; identity ≥30%; coverage ≥30%). Protein domains were searched against the Pfam database [29] of hidden Markov models using default parameters. Phage-like genes were predicted using Prophinder [30]. Clustered regularly interspaced short palindromic repeats (CRISPRs) were identified with CRISPRFinder [31]. Using the KAAS tool [32], we assigned protein-coding sequences to EC numbers when possible. Metabolic pathway reconstructions were then generated by matching EC numbers with KEGG pathways [33]. Orthology across whole chromosomes was determined using BLASTP reciprocal best hits (E-value ≤10^−10^; identity ≥30%; coverage ≥30%) in all-against-all comparisons of amino acid sequences in silico Molecular Cloning Genomics Edition (In Silico Biology). Synteny across whole chromosomes of *L. brevis* KB290 and ATCC 367 was visualized with the aid of GenomeMatcher software [34]. The draft genome sequence of *Lactobacillus iners* AB-1 (ADHG00000000) was obtained from GenBank. All KB290 genome sequence data were deposited in DDBJ/GenBank/EMBL and the accession numbers are as follows: AP012167 (chromosome), AP012168 (pKB290-1), AP012169 (pKB290-2), AP012170 (pKB290-3), AP012171 (pKB290-4), AP012172 (pKB290-5), AP012173 (pKB290-6), AP012174 (pKB290-7), AP012175 (pKB290-8), and AP012176 (pKB290-9).

### Tolerance of *L. brevis* strains to simulated gastric and intestinal juice

Tolerance to simulated gastric and intestinal environments was determined as previously described [35] with some modifications. Stationary phase cultures of *L. brevis* were inoculated (10% vol/vol) into simulated gastric juice (pH 2.85) containing 0.04% pepsin (Sigma), 0.1% mucin (Sigma), and 0.85% NaCl (Wako) and samples were incubated at 37°C for 3 h. Aliquots (25% vol/vol) were transferred to simulated intestinal juice (pH 7.0) containing 0.2% bile salt (Oxoid), 0.04% pancreatin (Wako), 0.04% trypsin (Wako), 0.1% mucin, 0.85% NaCl, and 0.6% Gifu Anaerobic Medium (GAM) broth (Nissui) and incubated at 37°C for an additional 7 h. For viable cell counts, we serially diluted each sample, poured it into MRS agar, and incubated it at 30°C for 2 days. The survival rate was calculated as the percentage of viable cells after treatment relative to the initial number of viable cells. We used SPSS (IBM, version 15.0J for Windows) for statistical analyses and considered *p*<0.05 as statistically significant.

### Aggregation assays

Bacterial strains were cultured in MRS medium at 30°C until late-logarithmic phase. The culture was then mixed vigorously for 30 s on a vortex-mixer, and fibrous-like aggregates became visible. After 5 min at room temperature, most of the aggregates precipitated and fell to the bottom of the tube. Once aggregated, cells did not redisperse. The aggregation phenotype was judged from the apparent lumpiness of bacterial aggregates.

## Results and Discussion

### General genome features

**Figure 1:**
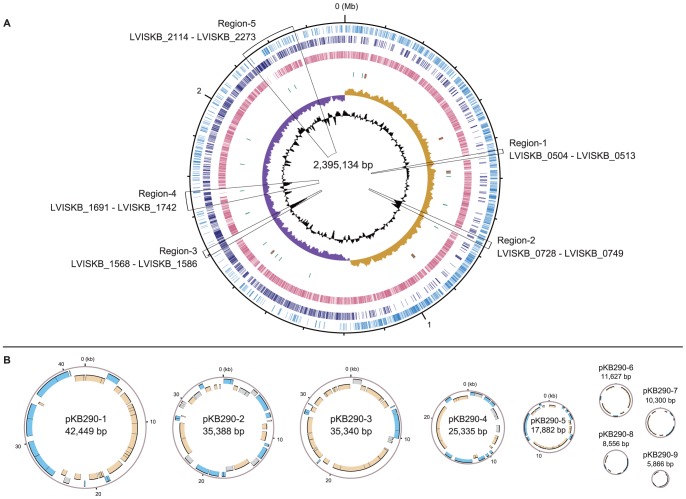
Circular representation of the KB290 chromosome (A) and nine plasmids (B). (A) From the outside in: Circles 1 and 2 of the chromosome show the positions of protein-coding genes on the positive and negative strands, respectively. Circle 3 shows the positions of protein-coding genes that have orthologs in *L. brevis* ATCC 367. Circle 4 shows the positions of tRNA genes (green) and rRNA genes (brown). Circle 5 shows a plot of GC skew [(G − C)/(G + C); khaki indicates values >0; purple indicates values <0]. Circle 6 shows a plot of G + C content (higher values outward). (B) The outer and inner circles of each plasmid represent genes on the positive and negative strands, respectively. The transposase genes are shown in gray.

The KB290 genome harbors a circular chromosome of 2,395,134 bp and nine distinct plasmids whose length ranges from 5.9 to 42.4 kb (Fig. 1). Among *Lactobacillus* species sequenced so far, this is the largest number of plasmids found in a single strain. The GC content of the chromosome is 46.1%, and the five rRNAs operons of 16S-23S-5S and 63 tRNAs encoding all 20 amino acids are similar to those in *L. brevis* ATCC 367 [3]. We predicted 2,391 protein-coding genes in the chromosome and 191 on the nine plasmids. Of the 2,582 protein-coding genes, 1,900 (73.6%) were assigned to a known function, 486 (18.8%) to conserved proteins of unknown function, and 196 (7.6%) to novel hypothetical proteins that are unique to KB290. We also identified 55 copies of transposase genes predominated by the IS30 family, more than found in ATCC 367. CRISPRs provide a defense against foreign genetic elements and have been observed in many lactic acid bacteria genomes [36]. KB290 contained one CRISPR (1,071,988–1,072,503), whereas ATCC 367 contained two. The KB290 CRISPR and one of the ATCC 367 CRISPRs were located at the same chromosomal locus, but their repeat sequences differed in type and number.

Table 2 summarizes the general features of the KB290 genome, and Table S2 shows some general features of both KB290 and ATCC 367. The concatenated sequences of ribosomal proteins from sequenced *Lactobacillus* species were used to investigate the phylogenetic relationships among these species, and KB290 and ATCC 367 belong to the same phylogenetic group at the species level (Fig. S1).

**Table 2:** General features of the *L. brevis* KB290 genome.

	Chromosome	Plasmid								
		pKB290-1	pKB290-2	pKB290-3	pKB290-4	pKB290-5	pKB290-6	pKB290-7	pKB290-8	pKB290-9
Size (bp)	2,395,134	42,449	35,388	35,340	25,335	17,882	11,627	10,300	8,556	5,866
GC content (%)	46.1	41.1	39.6	36.9	41.3	39.6	35.7	35.8	34.4	39.7
Protein-coding genes	2,391	37	34	34	25	29	10	10	6	6
Assigned function	1,787	20	23	23	14	19	3	6	2	3
Conserved hypothetical	437	14	8	8	9	7	2	1	0	0
Unknown function	167	3	3	3	2	3	5	3	4	3
rRNA operons	5	0	0	0	0	0	0	0	0	0
tRNA genes	63	0	0	0	0	0	0	0	0	0
Transposase gene	32	1	10	4	6	2	0	0	0	0
Prophage clusters	2	0	0	0	0	0	0	0	0	0

We observed no obvious differences in metabolic pathways in KB290 and ATCC 367 (Table S3), suggesting that both strains metabolize glucose via the pentose phosphate pathway [3]. We determined the fermentation substrate range for mono/oligosaccharides by the API 50 CHL assay (BioMérieux) and observed no difference between KB290 and ATCC 367 (data not shown), suggesting that both strains use the same glycolytic pathways. In ATCC 367, the chromosome encodes the highest number of transport proteins for the uptake and efflux of drugs and toxic compounds in sequenced lactic acid bacteria [3], [37], while in KB290, both the chromosome and plasmids encode such proteins (Table S4).

We previously reported that KB290's antibiotic resistance is intrinsic, was not acquired by lateral gene transfer, and is encoded on the chromosome [38]. In this study, using the MvirDB database, we surveyed the genes related to transferable antibiotic resistance and virulence factors in the KB290 genome, but we did not find any.

### Chromosome comparisons

The KB290 chromosome is about 104 kb larger than the ATCC 367 chromosome (Table S2). Both chromosomes shared 2,016 orthologous genes while 375 protein-coding genes were unique to KB290 and 169 were unique to ATCC 367 (Tables S5 and S6). The predicted amino acid sequence identity between the orthologs ranged from 25% to 100% (mean, 94%).

Of the 375 genes unique to KB290, 147 were assigned to novel hypothetical genes, 51 encode conserved proteins of unknown function, and 177 encode proteins of known functions (Table S5). Most of these genes are located in five large regions (Region-1 to -5) of the chromosome (Figs. 1 and S2). Region-1 (10 kb) contains two genes (LVISKB_0512 and LVISKB_0513) that encode putative cell surface proteins (Csc) that play a role in carbon source acquisition in *Lactobacillus plantarum* [39]. These Csc proteins might enhance utilization of plant materials. Region-2 (17 kb) and Region-4 (45 kb) are predicted prophage regions that contain integrase genes. Region-5 (137 kb) contains several genes predicted to be involved in sugar metabolism. Region-3 (19 kb) contains the cell wall-associated polysaccharide (CW-PS) biosynthesis gene cluster composed of 17 exopolysaccharide (EPS) and capsular polysaccharide (CPS) genes (Fig. S3 and Table S7), whose organization is similar to that of other sequenced lactic acid bacteria [40]–[43]. EPSs are linked to the host differential mucosal responses provoked by *Lactobacillus* [44] and also form a protective shield against host complement factors in the GI tract [45]. Glycosyltransferases are involved in the incorporation of polysaccharides into CW-PS biosynthesis [46]. pKB290-1 also contains two glycosyltransferase genes (LVISKB_P1-0027 and LVISKB_P1-0028) that are absent in ATCC 367, and the two strains have different surface structures (data not shown). The GC contents of Region-2 (42%) and Region-3 (39%) differ from the chromosomal average (46%) (Fig. 1), indicating that KB290 may have acquired these regions through lateral gene transfer.

On the other hand, of the 169 genes unique to ATCC 367, 110 were assigned to hypothetical genes and 59 to proteins of known functions (Table S6). Most are located in two large regions—Region-A and -B (>10 kb) on the ATCC 367 chromosome (Fig. S2). They include transposase genes (Region-A) and phage-related genes (Region-B). Alignment between the KB290 and ATCC 367 chromosomes also revealed several extensive genome-wide rearrangements that may have been generated by homologous recombination between mobile elements (Fig. S2).

### Plasmids

**Figure 2:**
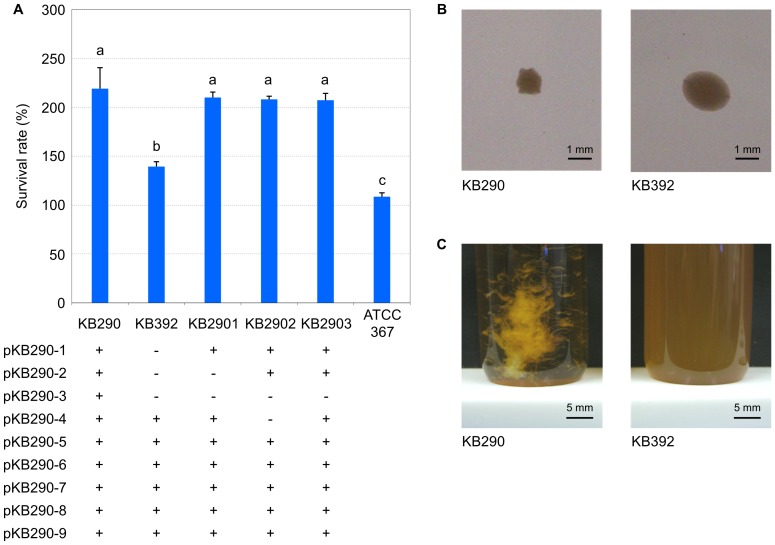
Properties of plasmid-cured strains. (A) Survival rates in simulated gastric and intestinal juice of KB290, ATCC 367, and various plasmid-cured KB290 strains. Results are expressed as mean + SD (n = 3). Different letters over the error bars indicate statistically significant differences (Tukey, *p*<0.05). Plus (+) represents the presence of the plasmid and minus (−) represents the absence of the plasmid. (B) Colony morphology of KB290 and plasmid-cured KB392. (C) Broth culture of KB290 and plasmid-cured KB392.

The nine plasmids in KB290 together carried 191 protein-coding genes, accounting for about 7% of the total. Of those, 173 (90.6%) of the predicted plasmid proteins had a significant similarity to known proteins and the highest similarity to those in other lactobacilli (Fig. S4). The remaining 18 genes showed no similarity to public database entries and so are likely unique to KB290. All of the plasmids had putative replication systems including *repABC* genes, which are essential for plasmid replication and stability. The replication proteins had diverged, as amino acid identities ranged from 24% to 100%. Thus, it is likely that KB290 has the highest number of mutually compatible *repABC* plasmids co-existing in a single bacterial cell. We derived several plasmid-cured strains but were unable to cure KB290 of all its plasmids (Fig. 2A), suggesting that the plasmids had different replication mechanisms. Further research is needed to confirm this. Many experiments have shown that host cells bear a cost for carrying a plasmid [47], [48]. Thus, the nine plasmids in KB290 may impart a wide range of unique features to the host, and the cost may be ameliorated by host-plasmid co-evolution. With the exception of pKB290-8, each plasmid contained several genes predicted to be involved in conjugation, presumptive CW-PS biosynthesis, the stress response, or other functions.

**Figure 3:**
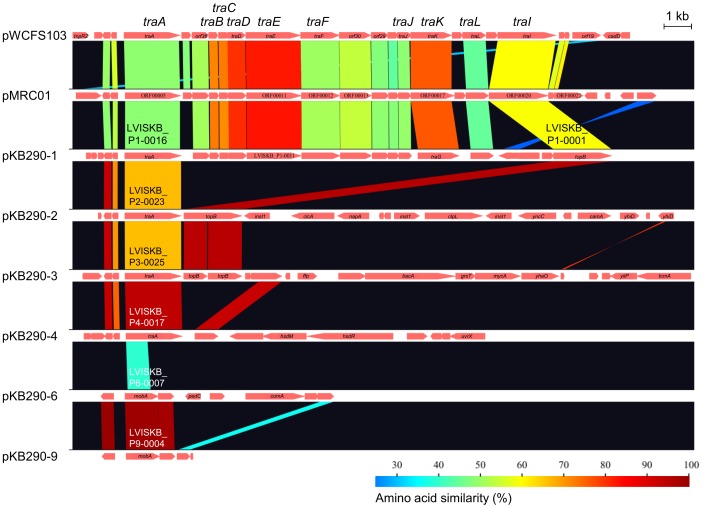
Conjugal transfer (*tra*) regions in the plasmids. Genes and their orientations are depicted with arrows.

Six plasmids (pKB290-1 to -4, -6, and -9) contain *tra* conjugation genes. The *tra* region of pKB290-1 showed high similarity and co-linearity with the *tra* regions of pWCFS103 in *L. plantarum* [49] and pMRC01 in *Lactococcus lactis* [50] (Fig. 3), suggesting that pKB290-1 could be self-transmissible in these species under certain conditions. The *tra* regions of five plasmids (pKB290-2, -3, -4, -6, and -9), on the other hand, have deletions, suggesting that additional functional genes are required for conjugation.

pKB290-1 also carries genes that are predicted to encode LVISKB_P1-0027 and LVISKB_P1-0028, glycosyltransferases probably involved in cell wall biogenesis (COG0463 and COG1215) [46]. Both have significant similarities with glycosyltransferase family 2 (Pfam PF00535) [51], [52]. This family is involved in the production of CW-PSs such as β-glucan with (1–3) linkages and other β-glycans [53]. Glycosyltransferase genes involved in CW-PS biosynthesis are also present in plasmids found in other genera, indicating that plasmid-borne glycosyltransferase genes are not unique to *Lactobacillus* [54], [55]. CW-PSs are also linked to the stress response, being involved in the formation of a protective shield against host innate defense molecules in the GI tract [45].

We also identified plasmid-encoded proteins that may be associated with the stress response (Table 3). Some may contribute to stressor removal, for example by encoding genes involved in multidrug resistance efflux mechanisms [56]. LVISKB_P2-0013 and LVISKB_P3-0008 encode putative multidrug resistance transporters, which possibly confer bile resistance. pKB290-3 carries genes encoding enzymes involved in non-ribosomal peptide or polyketide biosynthesis, which are needed for fatty acid synthesis (LVISKB_P3-0014), as well as genes predicted to encode cyclizing thioester proteins (LVISKB_P3-0015 and LVISKB_P3-0017), for the condensation of dipeptides with fatty acids, and for cysteine-dipeptide synthesis (LVISKB_P3-0016) [57], [58]. Such systems are found in other bacterial genera, including *Bacillus*, *Pseudomonas*, and *Streptomyces*, and in *L. lactis* plant isolates, where they may play a role in survival, defense, signaling, or adhesion [57], [59]. Many proteins for non-ribosomal peptide or polyketide biosynthesis are encoded on plasmids [60]. Thus, LVISKB_P3-0014 to -0017 may contribute to stress responses, enhanced under various environmental conditions.

**Table 3:** Putative stress response proteins encoded in plasmids.

Locus		No. of amino acids	Definition	Predicted function/role(s)
pKB290-1	LVISKB_P1-0027	344	Glycosyltransferase	Resistance to acid and bile
	LVISKB_P1-0028	654	Glycosyltransferase	Resistance to acid and bile
pKB290-2	LVISKB_P2-0013	458	Major facilitator superfamily permease	Adaptation to bile
	LVISKB_P2-0015	704	ATP-dependent Clp protease ATP-binding subunit ClpL	Resistance to acid and bile
pKB290-3	LVISKB_P3-0008	671	Drug resistance transporter, EmrB/QacA subfamily	Adaptation to bile
	LVISKB_P3-0014	552	Putative NRPS-encoding gene, *nrsF*	Survival, defense, signaling, or adhesion
	LVISKB_P3-0015	243	Putative NRPS-encoding gene, *nrsA*	Survival, defense, signaling, or adhesion
	LVISKB_P3-0016	1108	Putative NRPS-encoding gene, *nrsC*	Survival, defense, signaling, or adhesion
	LVISKB_P3-0017	331	Putative NRPS-encoding gene, *nrsB*	Survival, defense, signaling, or adhesion
pKB290-5	LVISKB_P5-0012	443	Glutathione reductase	Tolerance to oxidative stress
	LVISKB_P5-0015	51	Thioredoxin reductase	Tolerance to oxidative stress
	LVISKB_P5-0016	256	Thioredoxin reductase	Tolerance to oxidative stress
	LVISKB_P5-0018	107	Thioredoxin-like protein *ytpP*	Tolerance to oxidative stress
	LVISKB_P5-0019	104	Thioredoxin	Tolerance to oxidative stress
	LVISKB_P5-0020	93	Thioredoxin	Tolerance to oxidative stress
	LVISKB_P5-0022	155	DNA protection during starvation protein	Tolerance to oxidative stress and reduction of lipid oxidation
pKB290-7	LVISKB_P7-0006	155	DNA protection during starvation protein	Tolerance to oxidative stress and reduction of lipid oxidation

Even more vital to the general stress response are the chaperones involved in protein folding and protection, renaturation of denatured proteins, and removal of damaged proteins. Clp ATPases encoded by LVISKB_P2-0015 may play a similar role by degrading misfolded proteins and by being involved in protein reactivation and remodeling activities [61]. In contrast to heat shock proteins, Clp proteins seem to be particularly important for the fast response of lactobacilli when they encounter harsh conditions in the GI tract. In *Lactobacillus reuteri* ATCC 55730, ClpL is inducible under acidic shock [62] and has chaperone activity. Thus, LVISKB_P2-0015 may also contribute to acid and bile resistance.

Lactic acid bacteria show several adaptations to oxidative stress. LVISKB_P5-0012 encodes a glutathione reductase and LVISKB_P5-0015 to -0020 contain several genes that encode a thioredoxin system that catalyzes a wide spectrum of cellular redox reactions [63], [64]. Thus, pKB290-5 may confer microaerophilic growth condition tolerance to KB290, which is catalase-negative. In addition, LVISKB_P5-0022 and LVISKB_P7-0006, which might express DNA protection proteins during starvation, have a ferritin-like domain (PF00210) that could enhance tolerance to oxidative stress and reduce lipid oxidation [65], [66].

Stress-inducible proteins are likely to contribute to the survival of probiotic bacteria in the various environmental conditions they encounter in the host and also during industrial processes. Like *L. plantarum* and *Lactobacillus salivarius* [67], *L. brevis* is a multi-niche bacterium belonging to a group that contains regions of laterally transferred genes [40], [41]. Although we understand the distribution and function of some plasmids in the human gut mobile metagenome [68], little is known about the function of KB290 plasmids in the gut, and further research is needed.

### Properties of plasmid-cured strains

As mentioned previously, Table 3 lists the plasmid genes that could be involved in a stress response. The survival rate in simulated gastric and intestinal juice was considerably lower for KB392, which had been cured of three plasmids, than for KB290; the survival rate of ATCC 367 was also low (Fig. 2A). The survival rate for KB2901, however, which was cured of two plasmids (pKB290-2 and -3), showed no statistically significant difference from KB290 (Fig. 2A). In addition, all strains carrying pKB290-1 formed rough colonies with serrated borders on agar and aggregated in broth; KB392 formed smooth colonies on agar and did not aggregate in broth (Figs. 2B and 2C). These results suggest that pKB290-1 confers gastrointestinal tolerance and aggregatability on the KB290 phenotype. pKB290-1 encodes two glycosyltransferases (LVISKB_P1-0027 and LVISKB_P1-0028) that are not present in ATCC 367, and the two strains have different surface structures (data not shown). These genes may be involved in CW-PS biosynthesis and contribute to those phenotypic differences. The EPSs of *Lactobacillus rhamnosus* GG form a protective shield against host complement factors in the GI tract [45]. Glycosyltransferase is involved in the incorporation of polysaccharides into CW-PS biosynthesis [46]. The mechanism by which the KB290 CW-PS induces those changes is unknown, but it is possible that the CW-PS protects the cells from simulated gastric and intestinal juice and mediates cell–cell attachment for aggregation. Although our current understanding of the function and physiological relevance of CW-PS in KB290 is limited, glycosyltransferases in pKB290-1 may be responsible in part for strain-specific probiotic properties.

### Deep sequencing of KB290 culture stocks

Genomic stability of probiotic microorganisms used industrially is crucial for quality maintenance of commercial products [69]. The assessment of genetic and biological stability of bacteria can be generally performed by metabolic and biochemical tests and molecular analysis, such as random amplified polymorphic DNA and restriction fragment length polymorphism analysis followed by pulsed field gel electrophoresis for representative strains. Using those methods, we found KB290 to be highly stable over 15 years (data not shown). Conventional assessments, however, are not always able to detect low frequency variants that might affect the properties of the probiotic products.

**Figure 4:**
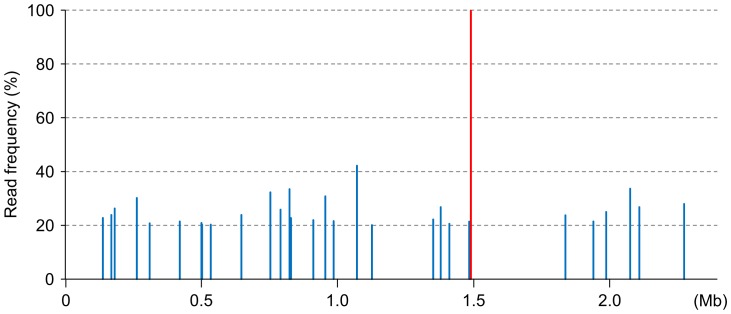
Distribution of mutation sites and minority variation sites in the chromosomes of periodic KB290 strains. The horizontal axis indicates the positions of the variant, respectively. Red and blue bars represent 2 mutation sites and 30 minority variation sites, respectively.

In this study, we surveyed four periodic KB290 strains for variants by performing deep sequencing with more than 1,000-fold chromosome coverage [70], [71]. We detected 37 minority variation sites with a single base substitution with a range between 20% and 58% read frequency (Fig. 4 and Table S8). We found 30 minority variant sites on the chromosomes and 7 on three of the plasmids. Of the chromosomal sites, 19 were non-synonymous and 5 were synonymous within protein-coding regions and 6 mapped to non-coding regions. Of the plasmid sites, one was non-synonymous and five were synonymous and within protein-coding regions while one was in a non-coding region. Twelve of the 20 total non-synonymous substitutions resulted in substitution of chemically similar amino acids.

In addition, we found three mutations with 99%–100% read frequency—two in non-coding regions of the KB290_1994 chromosome (positions 1,490,132 and 1,490,227) and one in a non-coding region of pKB290-3 of KB290_2006 (position 2,055) (Fig. 4 and Table S8). These mutations appeared only in the indicated stock and their effect on phenotype may be negligible.

We found a greater frequency of minority variation sites in the KB290_2009 than in the other stocks. We also found a greater frequency of base substitutions in the plasmids than in the chromosome and no base substitution hotspots (Fig. 4). The mechanisms behind these phenomena are so far unexplained.

**Figure 5:**
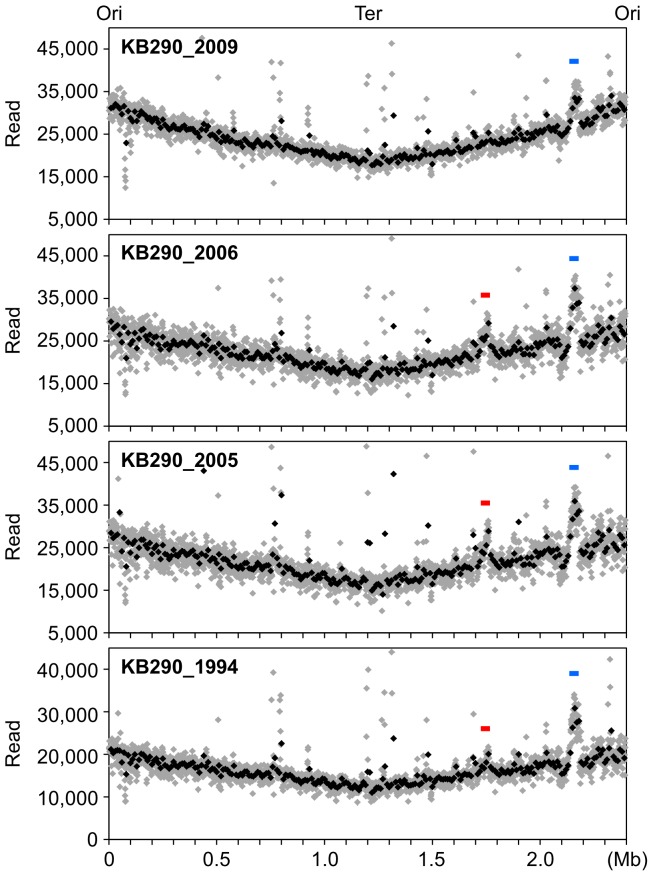
Mapping of the SOLiD reads on the KB290 chromosome. The SOLiD reads obtained from the four KB290 culture stocks were mapped to the KB290 reference genome. Number of reads mapped to the chromosome was plotted with a window size of 1 kb (gray diamonds) and the average of 10 kb (black diamonds). Red and blue bars represent the prophage clusters 1 and 2, respectively.

Mapping of the SOLiD reads of the four KB290 culture stocks on the entire KB290 genome revealed a nearly V-shaped distribution along the chromosome, where the number of reads at the replication terminus was about half that at the replication origin (Fig. 5), indicating synchronous bi-directional chromosome replication. We found more densely mapped regions around positions 1.74 Mb and 2.16 Mb, which contained prophage clusters. The prophage cluster 1 (43.6 kb) encoding 51 genes (LVISKB_1692 to LVISKB_1742) is a homolog of LBR48, isolated from a mitomycin-C-induced lysate of *L. brevis* C30 [72] (Fig. S5). The prophage cluster 2 (40.4 kb) includes 58 genes (LVISKB_2124 to LVISKB_2181) and is homologous to prophages LJ771 and LgaI, identified in probiotic strains *Lactobacillus johnsonii* [73] and *Lactobacillus gasseri* [74], respectively. LJ771 was shown to be a defective prophage, and the presence of LJ771 had no effect on the strain's growth or its gut persistence phenotype [73]. We have never seen lysis of KB290 cultures or decreases in their cell density. Thus, the prophage clusters found in the KB290 genome could be defective.

The commercial production of live probiotic bacteria requires the establishment of a quality assurance system that permits the efficient detection of contaminants and mutations over the long term. The methods we describe here for the detection of low-frequency variants throughout the genome provide a powerful and reliable approach for assessing and monitoring genomic stability.

## Supporting Information

Figure S1 **Genome-based phylogenetic analysis of KB290.** Phylogenetic relationships among the genomes of sequenced *Lactobacillus* inferred from 27 concatenated ribosomal protein amino acid sequences. The scale bar represents branch length. Bootstrap values are indicated at the nodes. Scale bar represents the number of substitutions per site. The unrooted tree was generated using NJplot.
(TIF)

Figure S2 **Synteny between the KB290 and ATCC 367 chromosomes.**
(TIF)

Figure S3 **Comparisons of the genomic location of the CW-PS gene cluster of KB290 with the corresponding location of other lactobacilli.** Genes and their orientations are depicted with arrows.
(TIF)

Figure S4 **Distribution of all protein-coding genes in KB290 based on the best BLASTP hits.**
(TIF)

Figure S5 **Genomic location of the KB290 prophage clusters and known prophages.** Genes and their orientations are depicted with arrows. (A) The prophage cluster 1. (B) The prophage cluster 2.
(TIF)

Table S1 PCR primers used for plasmid-specific detection.
(XLS)

Table S2 General features of *L. brevis* KB290 and ATCC 367 genomes.
(XLS)

Table S3 Metabolic pathways detected in *L. brevis* KB290 and ATCC 367.
(XLS)

Table S4 Transporters found in *L. brevis* KB290 and ATCC 367.
(XLS)

Table S5 The 375 chromosomal genes found in KB290 but not in ATCC 367.
(XLS)

Table S6 The 169 chromosomal genes found in ATCC 367 but not in KB290.
(XLS)

Table S7 The CW-PS cluster found in KB290.
(XLS)

Table S8 Mutations and minority variations in the genomes of four periodic KB290 strains.
(XLS)
