# Supplementary figures and images for: Genomic Analysis by Deep Sequencing of the Probiotic Lactobacillus brevis KB290 Harboring Nine Plasmids Reveals Genomic Stability
Source: PLoS One. 2013 Mar 27;8(3):e60521. doi: 10.1371/journal.pone.0060521 (PMC3609814; doi:10.1371/journal.pone.0060521)

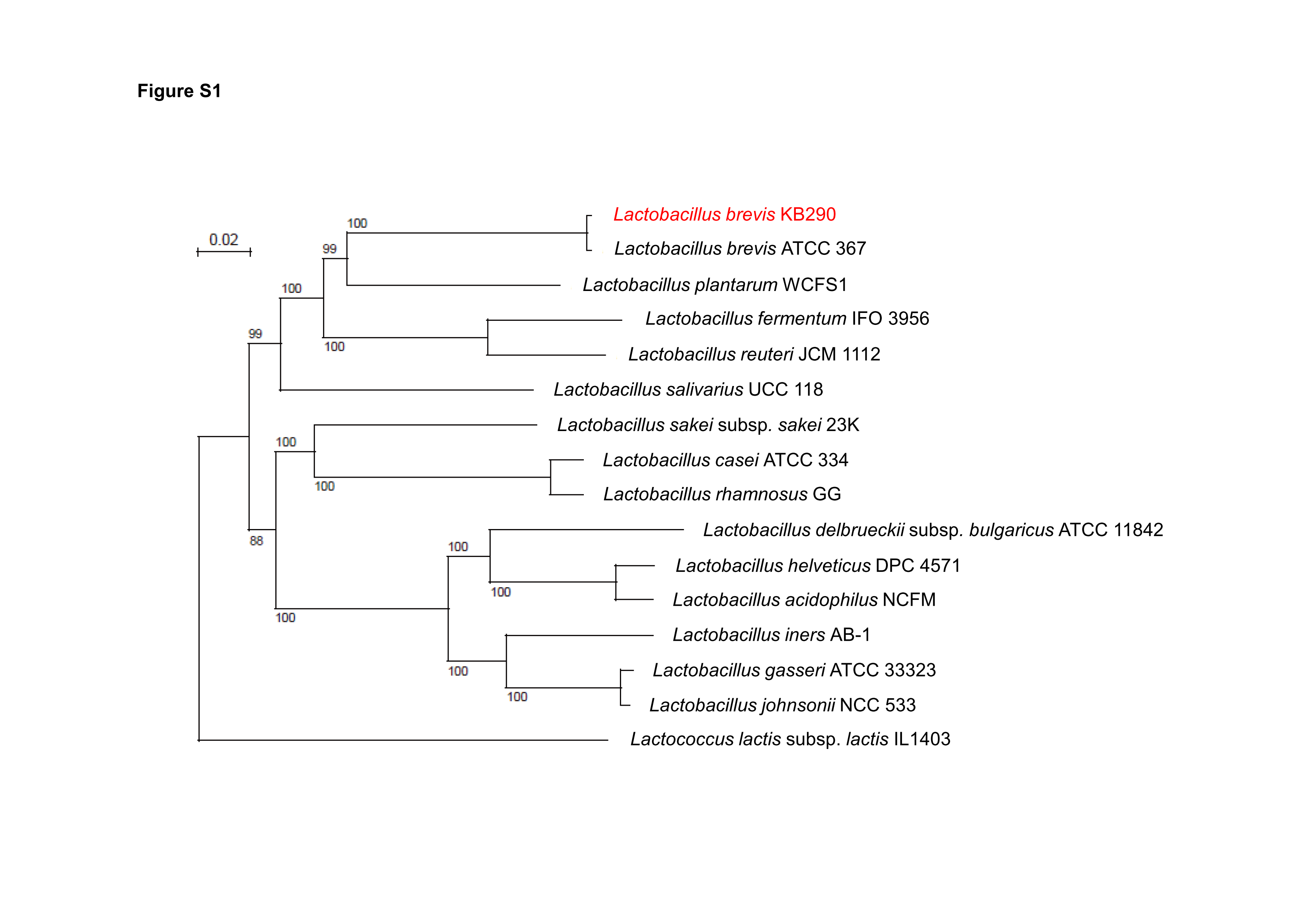

Supplement: Figure S1 — Genome-based phylogenetic analysis of KB290. Phylogenetic relationships among the genomes of sequenced Lactobacillus inferred from 27 concatenated ribosomal protein amino acid sequences. The scale bar represents branch length. Bootstrap values are indicated at the nodes. Scale bar represents the number of substitutions per site. The unrooted tree was generated using NJplot. (TIF) [file pone.0060521.s001.tif]

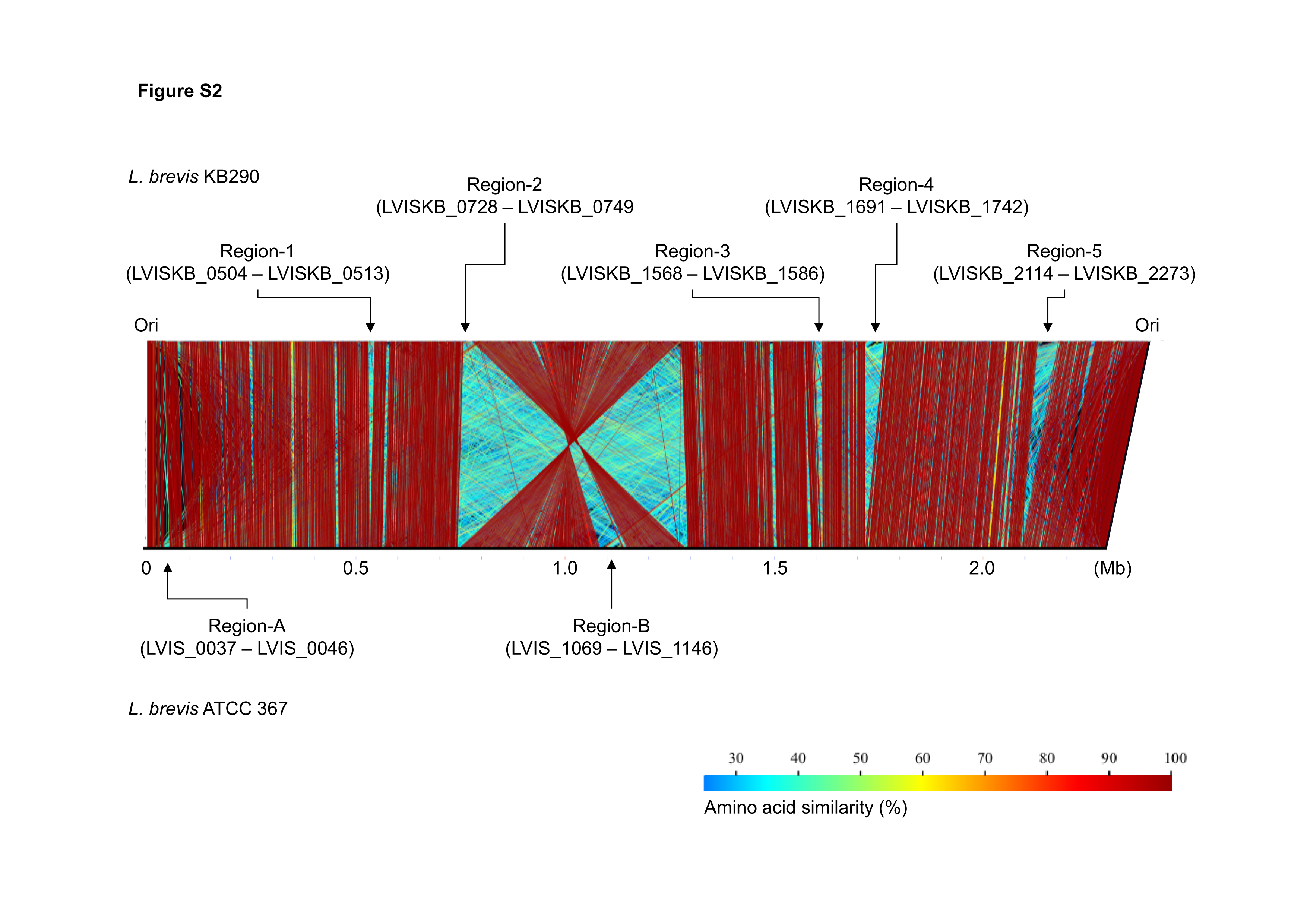

Supplement: Figure S2 — Synteny between the KB290 and ATCC 367 chromosomes. (TIF) [file pone.0060521.s002.tif]

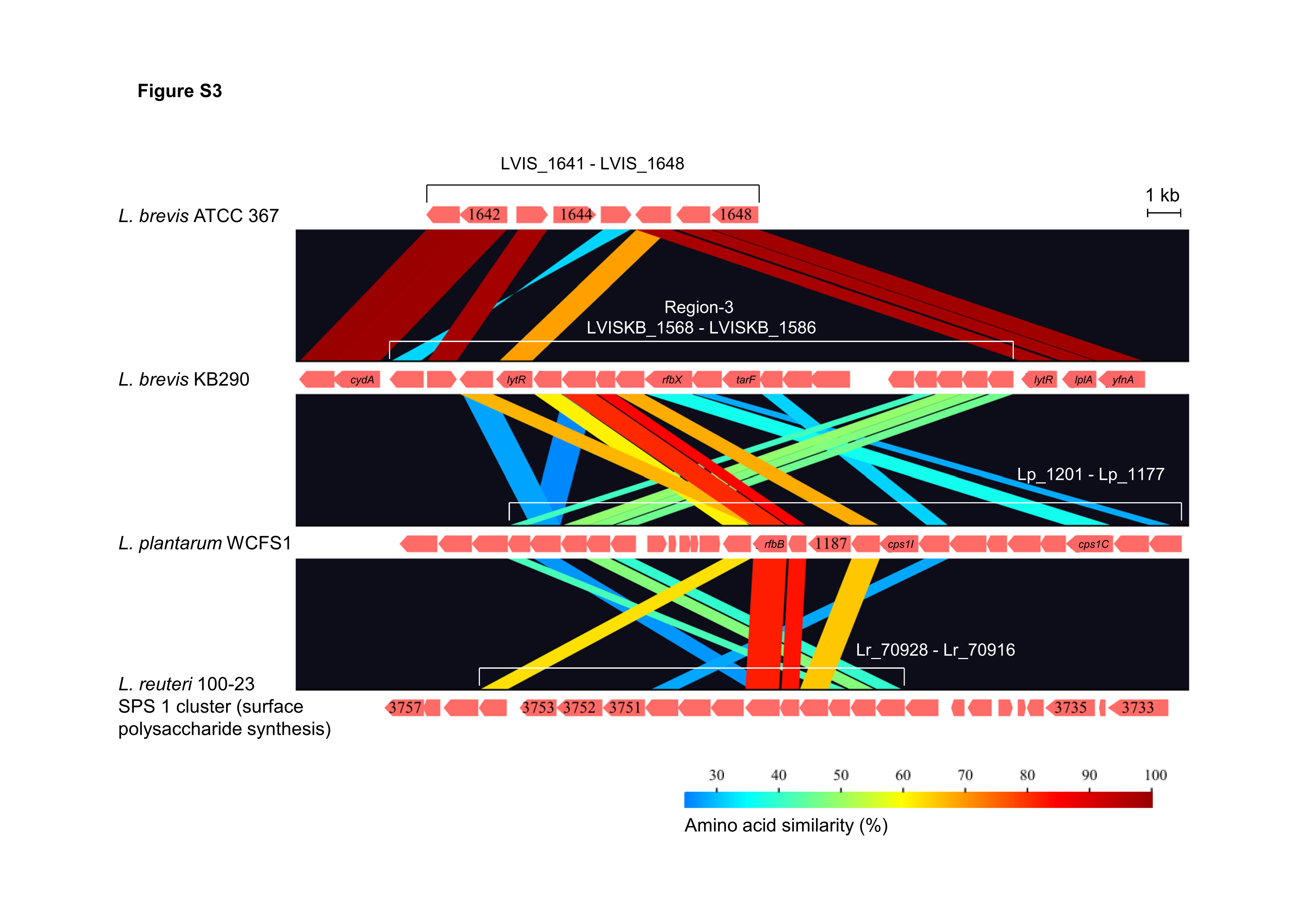

Supplement: Figure S3 — Comparisons of the genomic location of the CW-PS gene cluster of KB290 with the corresponding location of other lactobacilli. Genes and their orientations are depicted with arrows. (TIF) [file pone.0060521.s003.tif]

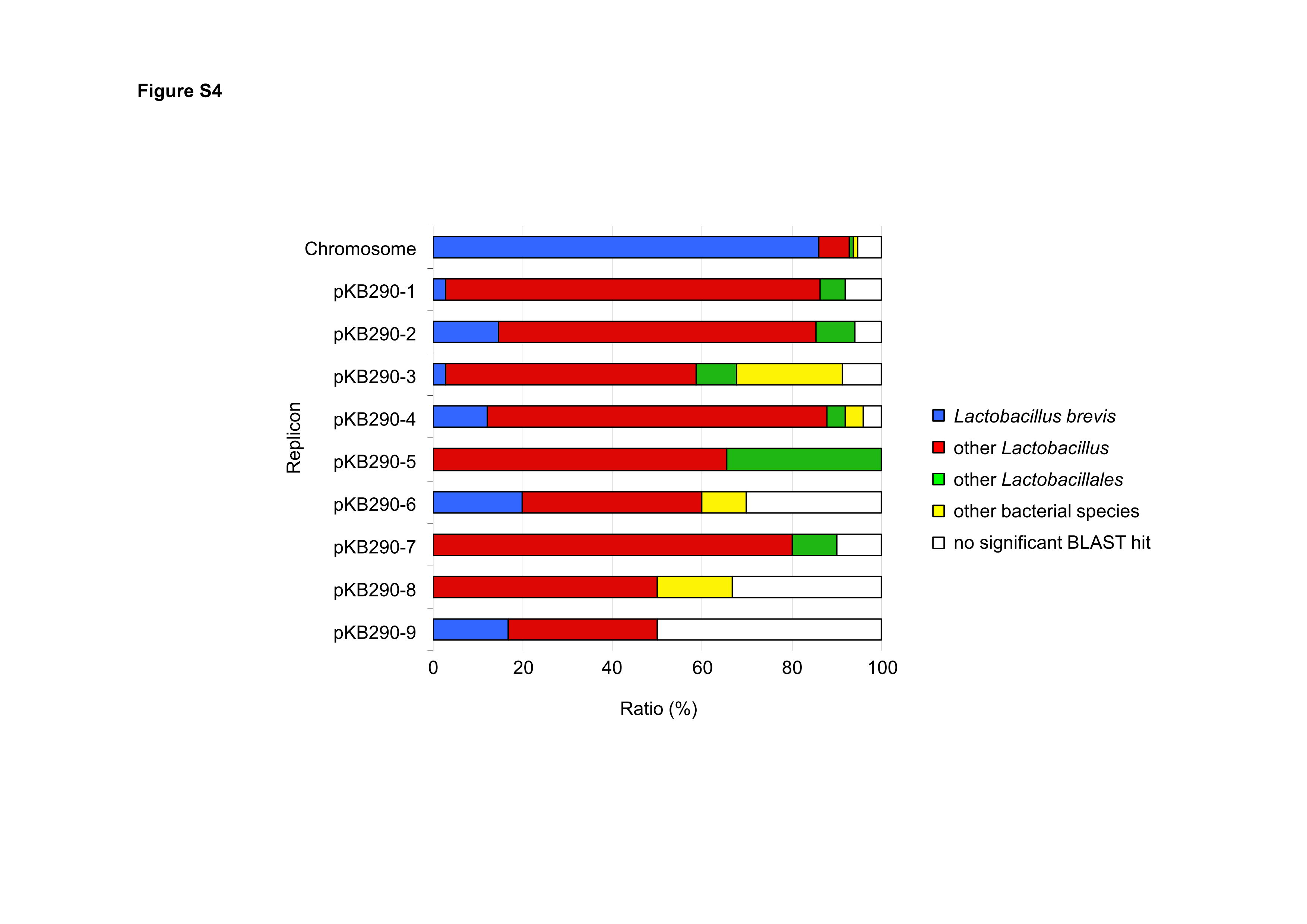

Supplement: Figure S4 — Distribution of all protein-coding genes in KB290 based on the best BLASTP hits. (TIF) [file pone.0060521.s004.tif]

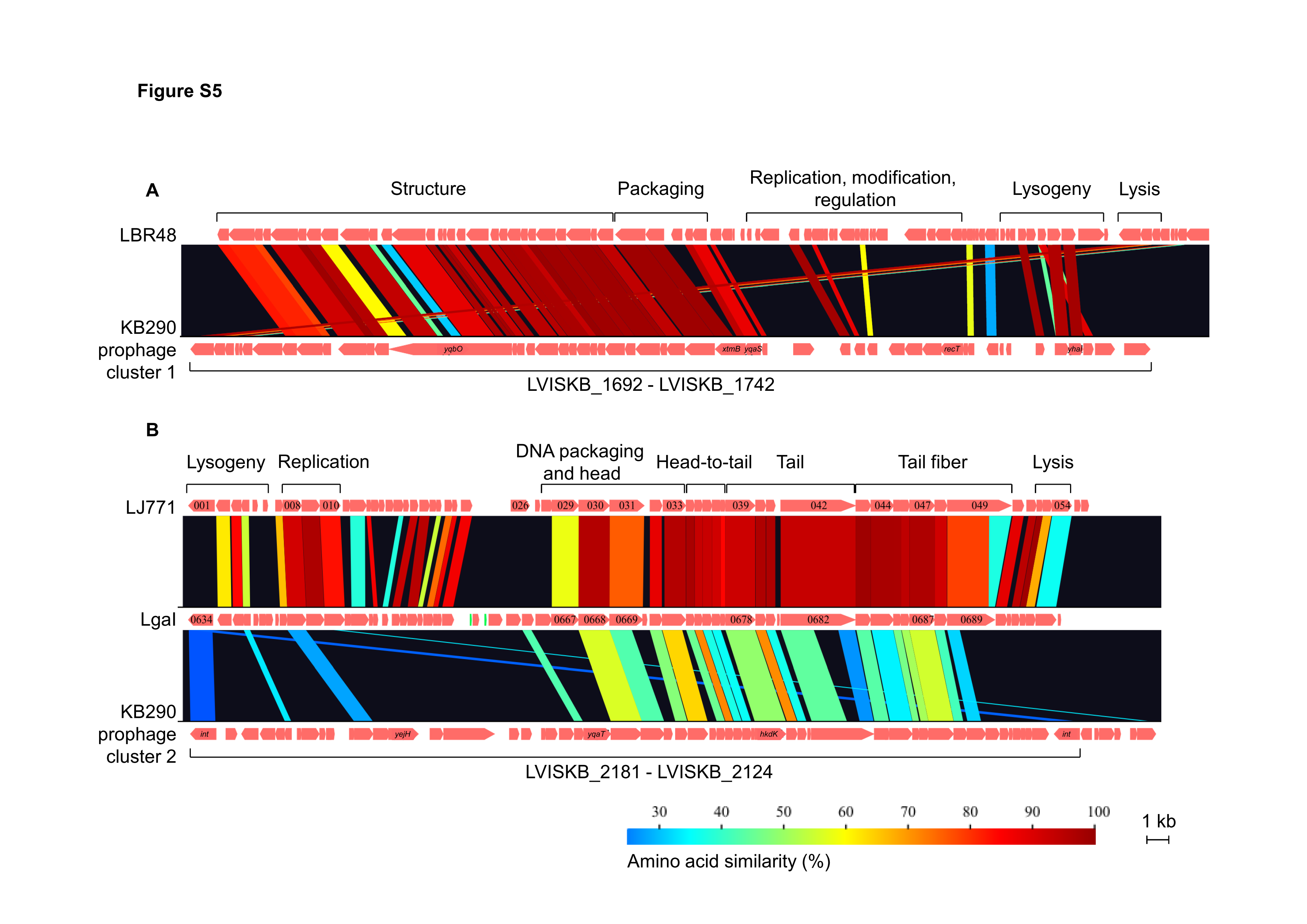

Supplement: Figure S5 — Genomic location of the KB290 prophage clusters and known prophages. Genes and their orientations are depicted with arrows. (A) The prophage cluster 1. (B) The prophage cluster 2. (TIF) [file pone.0060521.s005.tif]
